# The Cumberland Infirmary Inquiry

**Published:** 1920-10-02

**Authors:** 


### The Hospital

The Workers' Newspaper of Administrative Medicine and Institutional Life, Administration, National Insurance and Health.

No. 1791, Vol. LXIX. SATURDAY, OCTOBER 2, 1920. PRICE TWOPENCE

### THE CUMBERLAND INFIRMARY INQUIRY.

The psychology of governors of hospitals in general, and of the governors of Cumberland Infirmary in particular, is probably very much the same as that of the man in the street, who, when he scents a mystery, suddenly becomes acutely interested in a matter which had never stirred him before. The whole discussion at the quarterly meeting of the governors of Cumberland Infirmary, which is fully reported on page 9, seems to us to have been, to say the least, unnecessary, as the point at issue was capable of being decided by the use of common sense.

From what one can read between the lines, the trouble arose in the first instance when twenty-seven nurses and probationers signed a "round robin" presenting their grievances. The "round robin" is very much like the common or garden robin, which, when you treat it in a tactful way, becomes very friendly indeed.

There is nothing unusual at the present time about grievances in nursing circles, or, for that matter, in any other industrial circles. At a time when more work is required of everybody, and when there is less money to spend, all the world wants to do less work and have more money, and we are afraid that workers in hospitals of all classes cannot be excluded from the general rule. What is plain is that all grievances must be examined and dealt with carefully in the light of present-day conditions, and we believe that the Infirmary Committee dealt with the situation in the right spirit and made considerable concessions.

This move did not bring peace, and the help of an arbitrator was secured, who held an inquiry lasting for a day and a half, interviewing nurses and other members of the staff, and presented his report in due course.

So far so good. The first real trouble arose when the Committee began to make a mystery of this report, and were in doubt as to whether it should or should not be submitted to the governors. If in the first instance a few copies of the report had been duplicated, and it had been announced as open to the inspection of governors, we believe that very few would have wanted to see it, the majority probably being quite content that their representatives, the Committee, should deal with the matter in the manner appearing most expedient to them.

Instead of this, the same end was reached after a troublesome discussion at a general meeting, which, appearing in full in the newspapers, has set the public wondering whether there is not some widespread mismanagement at the infirmary; when, as we expect really is the case, merely some comparatively small trouble has arisen in departmental matters.

This is particularly unfortunate at a time when the hospital, like most other hospitals, is badly in need of money and is issuing an appeal. We think, however, that the storm will soon blow over in view of the wise decision of the quarterly court that a copy of Sir Napier Burnett's report, accompanied by his letter stating that the document is confidential, shall be open to the inspection of all interested governors.

If we may make a suggestion, we venture to propose that two or three of the leading governors of the infirmary, possibly accompanied by the Mayor of Carlisle or another prominent citizen, be asked thoroughly to examine the report and to write to the local Press stating that they have done so and that there is nothing in the management of the infirmary which could justify the public in withholding their sympathy and support.

We take it that the improvements suggested by

Sir Napier Burnett have a financial bearing on the matter, and this is one more reason for enlisting the whole-hearted co-operation of the district served by the infirmary. In any case, it is very evident that the air should be thoroughly cleared, in order that the infirmary may find itself in an unhampered position to go straight ahead on such lines of good management and beneficent work as are in accordance with the traditions of this excellent institution.

